# Perspectives of Immune Suppression in the Tumor Microenvironment Promoting Oral Malignancy

**DOI:** 10.2174/1874210601812010455

**Published:** 2018-06-20

**Authors:** Nobuo Kondoh, Masako Mizuno-Kamiya, Eiji Takayama, Harumi Kawati, Naoki Umemura, Yutaka Yamazaki, Kenji Mitsudo, Iwai Tohnai

**Affiliations:** 1 Department of Oral Biochemistry, Division of Oral Structure, Function and Development, Asahi University School of Dentistry, 1851 Hozumi, Mizuho, Gifu, 501-0296, Japan; 2 Department of Management and Information Studies, Chemistry Laboratory, Asahi University School of Business Administration, 1851 Hozumi, Mizuho, Gifu 501-0296, Japan; 3 Department of Oral Health Science, Gerodontology, Faculty of Dental Medicine and Graduate School of Dental Medicine, Hokkaido University, Kita-13, Nishi-7, Kita-ku, Sapporo, 060-8586, Japan; 4 Department of Oral and Maxillofacial Surgery, Yokohama City University Graduate School of Medicine, 3-9 Fukuura, Kanazawa-ku, Yokohama, 236-0004, Japan

**Keywords:** Immune suppression, Tumor Microenvironment (TME), T-Regulatory cell (Treg), Myeloid Derived Suppressor Cell (MDSC), Cancer-Associated Fibroblasts (CAFs), Oral malignancy

## Abstract

**Introduction:**

In order to survive, cancers control immune systems and evade immune detection using mediators consisting of immune checkpoint molecules and cellular systems associated with immune suppression.

**Methodology:**

During the development of cancer and chronic infections, the immune checkpoints and cellular components including regulatory T cells, myeloid derived suppressor cells and cancer associated fibroblasts are often enhanced as a mechanism of immune subversion and have therefore become very important therapeutic targets.

**Conclusion:**

In this review, we will discuss the complexity of immune-suppressive mechanisms in the tumor milieu of cancers, including oral malignancy.

## 1 INTRODUCTION

Accumulated evidence suggests that the progression and malignancy of tumors, including Oral Squamous Cell Carcinoma (OSCC), is not only promoted by the aggressive phenotypes of tumor cells, but is also regulated by the biological influences of the micro environments within the tumor tissue that affect the host’s immunological response [1]. Overexpression of immunosuppressive cytokines, such as IL-10, TGF-β and IL-1β can cultivate a Tumor Microenvironment (TME) conducive to evasion and tumor proliferation [2-4]. Cross talk between cancer and stromal cells including Cancer Associated Fibroblast (CAF) is mediated by IL-1β, and several other cytokine cascades [5]. The role of the immune suppressive mechanisms that are utilized by Myeloid-Derived Suppressor Cells (MDSCs) is essential for the suppression of T cell activity [6]. Co-evolution of both epithelial and stromal phenotypes contributes to carcinogenesis [7]. There are specific metabolic pathways that have been identified and shown to contribute to the enhancement of regulatory T (Treg) cells to suppress anti-tumor immunity [8]. The complex activity within immune checkpoint molecules include both inhibitory and co-stimulatory pathways that are located between Antigen Presenting Cells (APC) and effector T cells (or effector Treg), which can counteract T-cell mediated immunity due to exhaustion of the T cells by activated Treg and MDSCs [9]. The aim of this review is to summarize the immune suppressive networks that could therefore provide novel strategies to overcome T cell exhaustion [10], and to provide a clue for effective immunotherapeutics against OSCCs and other cancers [11].

## 2 IMMUNE CHECK POINT INHIBITORS IN CANCERS

The regulation of T cell activity requires at least two signals mediated by receptors. First, it requires T cell receptors which specifically engage peptides presented by Major Histocompatibility Complexes (MHCs) on Antigen-Presenting Cells (APCs). Second, T cell activation requires CD28 as a receptor for B7 family ligands that are located on APCs [12]. The aberrant expression of T cell co-stimulatory (B7 family) molecules in the tumor milieu has been attributed to the suppression of anti-tumor immunity. Cytotoxic T-Lymphocyte-Associated Protein-4 (CTLA-4; also known as CD152) is one of the first inhibitory receptors shown to be a suppressor of T cell response [13]. CTLA-4 is structurally similar to CD28 and binds to CD80 (B7-1) / CD86 (B7-2) on APCs (or tumor cells) at a higher affinity than CD28, which suggests an interference with T cell activation [14]. Overexpression of CTLA-4 is associated with a poor prognosis or higher clinical stages in nasopharyngeal carcinoma and breast cancer, respectively [15, 16]. CTLA-4 is the first immune check point targeted in the treatment of cancer. The blocking of CTLA-4 reduces tumor growth in murine models, including melanoma, and colon carcinoma [17, 18]. Both CD86 and CTLA-4 showed significant overexpression in the OSCC tissues with differentially methylated promoters [19]. Association of CTLA-4 gene polymorphism with OSCC has also been reported [20].

Another immunological checkpoint that facilitates tumor cell evasion involves the interaction between Programmed Death-1 (PD-1; CD279) and Programmed Death Receptor Ligand-1 (PD-L1; also known as CD274 or B7-H1). PD-1 is first identified as a type-I transmembrane receptor in a murine T-cell hybridoma clone undergoing Activation-Induced Cell Death (AICD) [21]. A recent study evidenced that CTLA-4 and PD-1 exert their effects at different stages of T-cell activation. CTLA-4 works mainly at the T cell priming stage in the lymph nodes, whereas PD-1 mainly promotes the cells in the periphery [22]. The expression of PD-1 on T cells has become one of the hallmarks of exhausted T cells. In multiple human tumors, a significant proportion of Tumor Infiltrating Lymphocytes (TILs) express PD-1 and are often associated with impaired CD8^+^ T cell function [23, 24]. In OSCCs, a correlation was shown between PD-L1 and tumor size and lymph node metastasis or other malignant phenotypes [25, 26]. Interestingly, however, the expression of PD-1 and PD-L1 in blood and lesion samples is elevated in both patients with OSCCs and pre-malignant actinic cheilitis [27]. These results suggest that the evasion of T cell activity by PD-L1 through signals of PD-1 is essential for the progression of OSCCs.

PD-1/PD-L1 interaction has been implicated in promoting the induction of Treg cells [28]; the blocking of PD-1 by antibodies suppresses TGF-β and retinoic acid-induced Treg conversion from naïve T cells, and treatment with a PD-1-blocking antibody in combination with a tumor vaccine was shown to reduce Treg infiltration into tumors *in vivo* [29]. Different immune checkpoint inhibitors including PD-1 and CTLA-4 can work synergistically due to non-redundant mechanisms. The synergistic blocking of PD-1 and CTLA-4 enhances and prolongs the efficacy of cancer vaccine to reject B16 melanoma cells by elevating the CD8+ / Treg ratio [30]. Studies have confirmed the drastic synergy effects of vaccines against tumor neoantigens with immune check point inhibitors. These studies have demonstrated that anti-CTLA4 treatments provide a highly effective immune therapy for melanoma [31, 32] and anti PD-1 treatments are highly advantageous for non-small cell lung cancers [33]. Positive results have also been observed for oral malignancies, whereby a combination of platinum-based chemotherapy and treatment with nivolumab, PD-1 monoclonal antibody resulted in a longer overall survival rate for patients with recurrent squamous-cell carcinoma of the head and neck [34].

Another potentially significant protein is the Inducible Co-Stimulator Ligand (ICOSL), also known as B7-H2. It shares homology with CD28, and similar to CTLA-4, it is induced following T cell activation [35]. In melanoma patients, the specific expansion of ICOS positive Tregs following high-dose IL-2 therapy correlates with worse clinical prognoses [36]. Acute myeloid leukemia patients exhibiting ICOSL positivity had significantly decreased survival [37]. The polymorphisms of the CTLA-4 in combination with ICOS seems to be possible predisposing factors for OSCC [38]. Interestingly, ICOS/ICOSL axis could have a dual effect to participate in anti-tumor T cell response as well as a pro-tumor response connecting with Tregs’ activity, therefore both antagonist and agonist antibodies targeting this pathway could be effective for cancer treatment. [39].

B7-H3 is part of the B7 family whose receptor remains unidentified. The *in vivo* expression of B7-H3 is reported in OSCC [40]. Contradictory findings show both co-stimulatory and co-inhibitory functions [41-44]. B7-H4 also represents a T cell-co-inhibitory molecule whose expression in the tumor milieu has been associated with poor prognosis of esophageal carcinoma [45], OSCC [46] and ovarian carcinoma [47, 48]. Tumor-Associated Macrophages (TAMs) express high levels of B7-H4, which is attributed to the presence of IL-6 and IL-10 [48]. Although, ligands on T cells are unidentified, the mobilization of stromal cells for immune evasion involving B7-H3 and B7-H4 could be similarly regulated by OSCCs.

V domain-containing Ig Suppressor of T-cell Activation (VISTA) is a member of the B7 family that bears homology to PD-L1 and is exclusively expressed within the hematopoietic compartment. VISTA overexpression on tumor cells interferes with protective antitumor immunity *in vivo* in mice [49] and human OSCCs [50].

## 3 REGULATORY T CELLS IN THE IMMUNE SUPPRESSIVE STRATEGIES IN CANCER

CD4^+^CD25^+^cells, Tregs, are identified as a naturally occurring CD4^+^ T-cell subset (about 5-10% of all peripheral T cells) by Sakaguchi and colleagues [51]. Most Tregs are generated in the thymus, and serve as regulators of overloaded immune reaction. It has been suggested that the Forkhead Box P3 (FoxP3) transcription factor represents an intracellular marker for Treg. There are also other markers, including CTLA-4, Glucocorticoid-Induced TNF Receptor (GITR), Lymphocyte Activation Gene-3 (LAG-3), and neuropilin [51]. FoxP3 transcription factor represses IL-2 production of Tregs, which also expresses IL-2 receptor (CD25). Therefore, for the survival of Tregs, an outer supply of IL-2 is essential [52]. In tumor tissues, many Tregs develop into effector Tregs which express CTLA-4, PD-1 or CCR6 at higher levels, and engage in the disruption of host immune systems including those in OSCC patients [53-55]. The elevated Foxp3 protein expression is a predictive factor for OSCC progression [56]. A higher frequency of double-labelled FoxP3^+^ and Toll-Like Receptor Protein 2 (TLR2)^+^ Tregs is observed within the immune cells of OSCC patients [57]. So, the blockade of Treg function using particular TLR ligands may be a potential target for therapeutic strategy.

## 4 MYELOID-DERIVED SUPPRESSOR CELLS IN THE TUMOR MICROENVIRONMENTS

MDSCs are a heterogenic cell population consisting of immature myeloid progenitors and precursors of granulocyte, macrophages and dendritic cells [58]. They are expanded in chronic, environmental, or tumor-associated inflammations and exert immune-suppressive effects, as well as supporting tumor growth and metastasis in a number of ways [59-62].

MDSCs have been described in tumor-bearing mice, in which they can be identified by the cell surface markers of CD11b and Gr-1 [63, 64]. They are further subdivided into two populations using Gr-1 isoforms Ly6G and Ly6C. The differential expression of these molecules distinguishes monocytic (M)-MDSCs from granulocytic (PMN)-MDSCs. M-MDSCs are CD11b^+^Ly6C^+^ Ly6G^low/-^; PMN-MDSCs are CD11b^+^Ly6C^-^Ly6G^+^ [65, 66]. Since humans lack an analogue of Gr-1, the human M-MDSCs are CD11b^+^CD14^+^CD15^-^IL4Ra^+^HLA-DR^low^CD33^+^; PMN-MDSCs are CD11b^+^CD14^-^CD15^+^HLA-DR^low/-^CD33^+^ [67].

Whereas MDSCs exist at a very low level in healthy peripheral blood, in cancer patients the population is expanded [6, 68]. The expansion of MDSCs is induced by pro-inflammatory cytokines including VEGF, IL-1β, IL-6, IL-17 and TNF-α. VEGF inhibits Dendritic Cell (DC) development *via* NF-kB activity while driving MDSC accumulation [69]. IL-1β is a potent inducer of MDSC accumulation and suppressive activity [70] and IL-6 is a downstream mediator of the IL-1β-induced expansion of MDSC [71]. While IL-17 is an inducer of MDSC, the effects may be mediated by IL-6 [72]. Granulocyte-Macrophage Colony Stimulating Factor (GM-CSF) is required for DC differentiation; however, high levels of GM-CSF induce MDSC accumulation [73]. TNF-α induces M-MDSC to promote immune tolerance [74]. Prostaglandin (PG) E_2_ is an inflammatory chemical mediator generated by Cyclooxygenase (COX)2. Many tumors, as well as tumor-infiltrating cells, produce PGE_2_. PGE_2_ promotes MDSC differentiation, while elimination of COX2 or PGE_2_ in tumor bearing mice reduces MDSC differentiation [75]. High Mobility Group Box (HMGB)1 is released from myeloid cells as a response to sepsis, infection, or arthritis. HMGB1 promotes the development of MDSCs from bone marrow progenitor cells, and resulted in the increased production of IL-10 [76]. Tumor-derived lactate is a product of the “Warburg effect” [77]. The lactate also develops MDSCs, and blunts immune surveillance [78, 79].

M-MDSCs are highly immunosuppressive and exert their regulatory activity mostly in an antigen-nonspecific manner. In contrast, PMN-MDSCs are moderately immunosuppressive and suppress immune responses mainly by antigen-specific mechanisms [80]. Both subsets of MDSCs are expanded systemically in a number of human malignancies. Renal cell carcinoma, colorectal cancer, and hepatocellular carcinoma are associated with PMN-MDSC, while glioblastoma, ovarian cancer and multiple myeloma are associated with M-MDSC. Metastatic melanoma and non-small cell lung cancer are associated with both types of MDSCs [81]. Expression of VISTA and MDSC markers are also correlated with the poor prognosis in primary OSCC [50].

In order to compare the immune-modulatory effects of OSCCs in primary and advanced stages, we have established a metastasized model (L5-11cells) representing more malignant phenotypes than the parental OSCC (Sq-1979 cells) when implanted in the syngeneic mice [82]. Our results revealed that it is metastasized L5-11 cells, not early-stage Sq-1979 cells that predominantly induce PMN-MDSCs in tumor-bearing mice [83]; this is not simply reflecting the differential tumor size, since even the larger amount of inoculation of Sq1979 cells could not induce the MDSC cells in the mice. We have also confirmed that the quantity of IFN-γ produced by stimulated spleen cells is significantly suppressed in mice implanted with Sq-1979 and L5-11 cells; however, the production of IL-10 is significantly elevated in mice implanted with Sq-1979 while markedly suppressed in L5-11 cell-implanted mice [84]. Thus, our results demonstrated that unlike primary OSCC cells, the metastasized OSCC cells have acquired immunomodulatory functions mediated by PMN-MDSCs.

MDCSs utilize multiple suppressive mechanisms to promote tumor expansion. They directly suppress T cells by starving them of amino acids, inducing apoptosis, and reducing their intracellular signaling. MDSC-inducing immune suppressions are mediated by Arginase (ARG)1, inducible NOS (iNOS), NO [85-87], sequestration of cysteine, and decreased expression of E-selectin [88, 89]. In the case of OSCC patients, CD14^+^/CD16^+^ monocyte-derived macrophages, a potential subpopulation of MDSCs, are increased in circulating peripheral blood [90]. The effect of MDSCs on the systemic immunity of advanced-stage OSCC patients may be the cause of the increased proportion of CD57^+^ T cells [91], or the higher levels of Th2 in combination with relatively lower levels of Th1 cytokines [92].

## 5 CANCER-ASSOCIATED FIBROBLAST AS A MEDIATOR OF IMMUNOSUPPRESSION

Several lines of evidence have demonstrated that stromal cells surrounding tumor cells play important roles in developing tumor tissues. High-grade invasive OSCC cells specifically induce PD-L1 on dendritic cells in the TME [93]. In head and neck cancers, CAFs have been suggested to promote angiogenesis, invasion metastases, and Treg induction *via *several cytokines including HGF, IL-33, IL-6, CCL2, CCL7, TGF-β1, VEGF and TNF-α [2]. CAF-educated macrophage progenitor cells reduce T cell proliferation *via* TGF-β1, IL-10 and ARG1, suggesting that CAFs can induce protumoral TAMs in the tumor immunosuppressive microenvironment [94]. In OSCC tissues, CAFs are divided into 3 grades on the basis of the expression of alpha smooth muscle actin, and the CAFs in the higher grade promote CD163 positive macrophage which is associated with poor prognosis [95]. Constitutive production of IL-1β from OSCCs enhances IL-6 production of CAFs [4]. IL-6 and GM-CSF, produced by pancreatic CAFs, are important mediators of MDSC differentiation [96]. Our results using a mouse OSCC model suggest that the immune-suppressive function of mesenchymal stromal cells is specifically enhanced by humoral factor(s) from primary OSCC cells [97], which, however, do not induce any MDSCs in the tumor-bearing mice [84]. Therefore, immune-modulatory functions of OSCC that are dependent on mesenchymal stromal cells and MDSCs can be uniquely regulated according to their malignant stages.

## 6 THERAPEUTIC APPROACH OF ORAL PRE-CANCEROUS DISORDERS

The immune system plays an important role in the development and progression of OSCCs and other cancers. As already mentioned, recent cancer immunotherapies have targeted the molecules in the tumor microenvironment of immune check point inhibitors including CTLA4 [17, 18, 29-32], PD-1 [29, 33, 34] or ICOS [39].

The histopathology of oral malignancies has shown that some OSCCs develop from Oral Potentially Malignant Disorders (OPMDs) in a complex tissue microenvironment. Such examples of OPMDs include: Oral Erythroplakia (OE), oral leukoplakia (OLK), oral lichenplanus (OLP) [98, 99]. Of these, it is, OLP that is most remarkable histologically due to its high number of lymphocytes. There are various therapeutic attempts being performed that aim to re-organize the microenvironment components of OLP. These treatments commonly include Thalidomide, an immune stimulant that inhibits TNF-α and stimulates T cells, M1 macrophage and NK cells [100, 101]. Another immune-stimulant, curucumin [102] has also proven effective in OLP treatments.

The use of steroids is another mainstay treatment for OLP because of their potential to inhibit immune cells that reduce cytokine production [103]. Other therapies recruited in the treatment of OLP employ the use of mycophenolate, a potent suppressor of B and T cells and chemokines [104], and pimecrolimus, which is a calcineurin inhibitor that has a suppressive effect on cytokines [105].

## CONCLUSIONS AND FORESIGHT

As summarized in Fig. (**1**), there are several mediators involving specific cytokines and cellular systems associated with tumor evasion of host immune systems. Our results demonstrate that the immunosuppressive efficacy of OSCC milieu is developed in a stepwise manner depending on the stages of OSCCs [84]. In the early stage, mesenchymal stromal cells (*i.e*., CAF) could be a unique effector; humoral factor(s) from OSCC cells force CAF to exert immune suppression *via *the direct cell contacts to effector T cells [97]. Potentiated CAF could also affect other immune-suppressive mediators such as Treg, TAM and MDSCs. In the advanced stage, MDSCs could possibly be a major conductor of immune-suppression [83]. In the TME, the effector Treg, harboring CCR6, PD-1 and CTLA-4, could be mobilized by antigens and/or chemokines secreted from OSCC cells. As shown in Fig. (**1**), there are several cytokines, immune-check points, and cellular components among the TME of OSCC. However, how OSCCs differentially utilize the immune modulatory aspects involving CAFs, MDSCs and several other factors is not fully understood. Further elucidation of the regulatory pathways structured by tumor-host interactions could identify important therapeutic targets in OSCC development.

## Figures and Tables

**Fig. (1):**
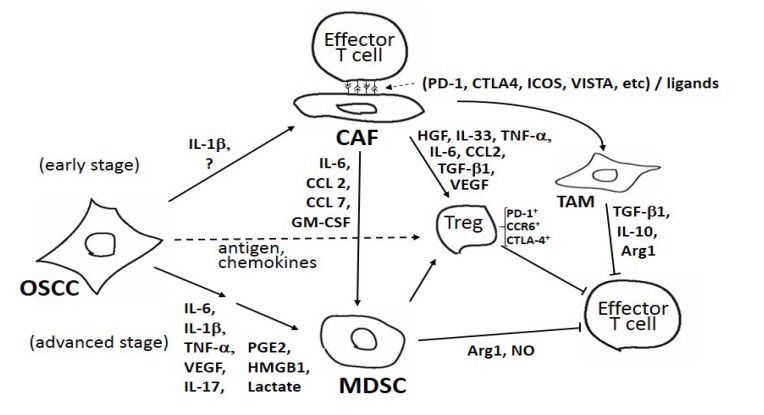
A schematic illustration of the cellular components and factors responsible for the immune-suppressive mechanisms in the tumor micro environment. Cellular components including Oral Square Cell Carcinoma (OSCC), Myeloid Derived Suppressor Cell (MDSC), Cancer Associated Fibroblast (CAF), Tumor Associated Macrophage (TAM), regulatory T cells (Tregs) and effector T cells, interact mutually using soluble factors, including Interleukins (ILs), C-C Chemokine Ligands (CCLs), Tumor Necrosis Factor (TNF)-α, Tumor Growth Factor (TGF)-β, High Mobility Group Box (HMGB) 1, Granulocyte Macrophage-Colony Stimulating Factor (GM-CSF), Vascular Endothelial Growth Factor (VEGF), Arginase (Arg) 1, Prostaglandin (PG) E2, Nitric Oxide (NO) and lactate. CAFs and Tregs can also interact directly with effector T cells *via* receptor-ligand interactions, including Programmed cell Death (PD)-1, Cytotoxic T-Lymphocyte-Associated Protein (CTLA)-4, Inducible Co-Stimulator Ligand (ICOSL), V Domain-Containing Ig Suppressor of T-Cell Activation (VISTA) and C-C Chemokine Receptor (CCR) 6.
